# A high-calorie diet exacerbates lipopolysaccharide-induced pneumonia by promoting acetate-mediated macrophage polarization via the HDAC9/10–HIF-1α–glycolysis axis

**DOI:** 10.3389/fimmu.2025.1614768

**Published:** 2025-09-26

**Authors:** Qianqian Li, Hui Liu, Chen Bai, Lin Jiang, Chen Su, Xueying Qin, Tiegang Liu, Xiaohong Gu

**Affiliations:** ^1^ School of Traditional Chinese Medicine, Beijing University of Chinese Medicine, Beijing, China; ^2^ School of Traditional Chinese Medicine, Shandong University of Traditional Chinese Medicine, Jinan, China; ^3^ Institute of Chinese Medicine Epidemic Disease, Beijing University of Chinese Medicine, Beijing, China; ^4^ Dongzhimen Hospital, Beijing University of Chinese Medicine, Beijing, China

**Keywords:** high-calorie diet, pneumonia, macrophage, hypoxia inducible factor-1α, glycolysis

## Abstract

Lung macrophage polarization imbalance is an important cause of aggravated pulmonary inflammation. The gut microbiota metabolites short-chain fatty acids (SCFAs) are an important regulator of macrophage polarization. A high-calorie diet has been shown to aggravate pneumonia and delay recovery, especially in children. However, the underlying mechanisms remain unclear. Our previous studies showed that a high-calorie diet can disrupt the gut microbiota structure and SCFA metabolism to aggravate LPS-induced lung inflammatory damage in juvenile rats. In this study, we investigated whether pneumonia aggravated owing to a high-calorie diet is associated with SCFA-driven macrophage phenotype changes in distal lung tissues and related mechanisms. Our data revealed that a high-calorie diet significantly aggravated pulmonary inflammatory injury in juvenile mice with LPS-induced pneumonia and also increased lung tissue M1-like (CD206-CD86+)/M2-like (CD206+CD86-) macrophage polarization imbalance. We found that a high-calorie diet decreased SCFA levels in mouse stool, serum, and lung tissues, which was most pronounced for acetate. Furthermore, we found that acetate reduction mediated by a high-calorie diet exacerbated M1-like (CD206⁻CD86⁺)/M2-like (CD206⁺CD86⁻) macrophage polarization imbalance in the lung tissue of pneumonia model mice and was associated with inhibiting histone deacetylase (HDAC), rather than G-protein-coupled receptor 43 (GPR43) signaling. More critically, we found that acetate supplementation had the most significant impact on HDAC9 and HDAC10 in the lung macrophages of pneumonia model mice fed a high-calorie diet. Furthermore, overexpression of *Hdac9* and *Hdac10* significantly attenuated the improvement effects of acetate on lung tissue M1-like (CD206^-^CD86^+^)/M2-like (CD206^+^CD86^-^) macrophage polarization in pneumonia model mice fed a high-calorie diet, and this mechanism was associated with the HIF-1α–glycolysis axis. Taken together, we demonstrated that a high-calorie diet could cause acetate levels to decrease in mice with LPS-induced pneumonia. This decrease in acetate was associated with a diminished inhibitory effect on HDAC9/10, potentially contributing to upregulation of HIF-1α expression and increased glycolysis. These changes may be linked to an imbalance in M1-like (CD206^−^CD86^+^)/M2-like (CD206^+^CD86^−^) macrophage polarization and aggravate lung tissue inflammatory injury. Our findings show that acetate supplementation may be a potential treatment strategy to prevent and treat pneumonia and other infectious diseases.

## Graphical Abstract

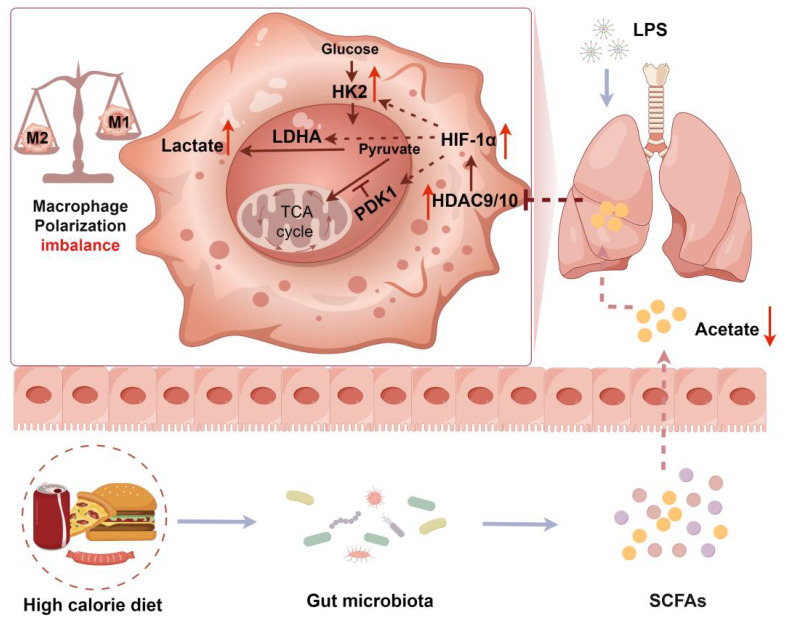

## 1 Introduction

Pneumonia is a common acute respiratory tract infection mainly caused by bacteria, viruses, and fungi. Pediatric pneumonia is the most common cause of hospitalization among children in developed countries (1) and is a leading cause of child mortality in developing countries (2, 3). In the past 2 years, the incidence of pediatric pneumonia has been increasing steadily (4, 5). Therefore, pediatric pneumonia remains an important clinical and public health concern (6). Consumption of sugary drinks and fast food is very common in children owing to child, peer, parental, and school factors (7). These foods contribute a large amount of calories but have low nutrient content. Related studies have found that the occurrence of respiratory tract infection is related to poor dietary habits (8). A high-calorie diet will activate innate immunity and impair adaptive immunity, resulting in chronic inflammation and decreased host defenses against respiratory pathogens (9). Nguyen et al. (10) found that a high-fat diet induced more severe lung inflammation in obese mice after treatment with LPS. Chen et al. (11) found that a high-fat diet induced more severe lung injury in obese mice after exposure to particulate matter ≤2.5 micrometers in diameter. Available reports demonstrate that a high-calorie diet exacerbates LPS-induced lung inflammatory damage (12, 13). These findings demonstrate the important role of diet in the progression and outcome of pediatric pneumonia. However, in-depth studies have not been conducted on the specific mechanisms by which a high-calorie diet aggravates pediatric pneumonia.

The gut microbiota represents an important factor in the maintenance of intestinal homeostasis that affects health and disease in the host (14). In turn, the effects of the gut microbiota on host health are mainly mediated by the metabolite SCFAs (15). The gut produces approximately 500–600 mM SCFAs every day, which are predominantly acetate, propionate, and butyrate. The specific SCFAs are determined by the diet, type and quantity of microbiomes, and residence time in the gut (16). The SCFA content is highest in the gut and can reach distal organs via the systemic circulation to exert immunomodulation effects. SCFAs are considered important regulators of host defenses and inflammation in infection (17) and can alleviate lung injury during respiratory tract infection (18, 19). Tian et al. (20) found that lung SCFA levels determine the baseline lung immunity level at rest and may be vital for immune responses in lung injury. Trompette et al. (21) found that a high-fiber diet increased SCFA levels in mice and decreased lung allergy whereas a low-fiber diet had the opposite effects. At present, an increasing body of evidence shows that SCFAs participate in the host defense against respiratory tract infection (22) and affect the severity of acute respiratory tract disease (23). The main mechanisms of action of SCFAs include inhibiting histone deacetylase (HDAC) to regulate gene expression (24), activating signal transduction through G protein–coupled receptors (GPCRs) (25), and participating directly in cell activation via metabolism (22, 26). Meanwhile, research has indicated that HDAC10 is a critical factor in the development of pulmonary inflammation (27). The study also found that HDAC9 plays a critical role in macrophage inflammation (28).

Lung macrophages are the first line of defense against airborne pathogens and are critical in maintaining lung immune equilibrium. An increasing body of evidence shows that macrophages participate in the pathogenesis of acute lung injury, acute respiratory distress syndrome, and other lung inflammatory diseases (24, 29, 30). Lung macrophage polarization imbalance is an important cause of aggravated pulmonary inflammation as well as the occurrence and progression of acute lung injury (31). Diet can affect macrophage polarization; some studies have shown that a high-fat diet promotes liver M1 macrophage polarization (32, 33) and colonic M1 macrophage polarization as well as elevation of pro-inflammatory factors (34). According to reports, the macrophage phenotype is intimately associated with metabolism because M1 macrophages are highly dependent on glycolysis and M2 macrophages mainly rely on oxidative phosphorylation (35). HIF-1α activation can promote macrophage glycolysis and is associated with polarization toward the M1 pro-inflammatory phenotype (36). SCFAs can affect the function of innate immune cells by rewiring the metabolism of macrophages, monocytes, and neutrophils (37). SCFAs produced by the gut microbiota represent an important regulator of macrophage polarization (37).

Our previous studies demonstrated that a high-calorie diet could induce gut dysbiosis, decrease gut SCFA levels, and aggravate pulmonary inflammatory injury in juvenile mice with induced pneumonia (12, 13). However, it remains unclear whether the mechanism via which a high-calorie diet aggravates pneumonia is associated with SCFA-mediated macrophage polarization. Therefore, we constructed a high-calorie diet pneumonia juvenile mouse model in this study to observe the effects of a high-calorie diet on lung macrophage polarization and SCFA levels in juvenile pneumonia model mice. SCFA refeeding and construction of *Hdac*-overexpressing mice were used to examine the relationship between pneumonia aggravated by a high-calorie diet and SCFA-mediated macrophage polarization, as well as their mechanisms.

## 2 Materials and methods

### 2.1 Chemicals and reagents

LPS was obtained from Sigma (St. Louis, MO, USA). MS Mouse IL-1β/IL-6/IL-10/TNF-α ELISA kits were supplied by Elabscience Biotechnology Co.,Ltd (wuhan, China). cDNA Reverse Transcription Kits were obtained from Thermo Fisher Scientific (Waltham, MA, USA). TRIzol reagent was supplied by Invitrogen (Austin, TX, USA). SYBR Green PCR Master Mix were purchased from Bio-Rad Laboratory (Richmond, CA, USA). BCA protein assay kit was purchased from Beyotime Biotechnology Co., Ltd (shanghai, China). The antibodies used in this study were listed in the **Supplementary Table S1**.

### 2.2 Animal study

The animal experimental procedures and animal care techniques were approved by the Institutional Animal Care and Use Committee of Beijing University of Chinese Medicine (BUCM-2023022401-1101). C57BL/6N mice (4-week-old male, 13 ± 2 g) were obtained from Vital River Laboratory Animal Technology, with license no. SCXK (Jing) 2021-0006 (Beijing, China). Mice were housed in the animal laboratory of Beijing University of Chinese Medicine. All mice had free access to water and food. Mice were randomly separated into four groups (*n* = 6): (1) normal control group (N), (2) pneumonia group (P), (3) high-calorie diet group (G), and (4) high-calorie diet combined with pneumonia group (GP). As shown in **Table 1**, the mice from the G and GP groups were administered the high-calorie fodder. Mice from the N and P groups were administered the mouse maintenance fodder. Starting on the fourth day of the experiment, the mice in the P and GP groups were exposed to an aerosol of LPS solution (0.5 mg/mL) for 30 minutes per day via inhalation. The N and G groups were exposed to an aerosol of sterile water for the same duration. Mouse maintenance fodder and high-calorie fodder were obtained from SPF Biotechnology Co., Ltd. The ingredients and nutrition are shown in our previous report (12). On the seventh day of the experiment, all mice were anesthetized by intraperitoneal injection of 2% sodium pentobarbital (0.1 mL/10 g). Blood and lung tissue samples were collected for further analysis. The lung tissues of the mice were rinsed with physiological saline to remove residual blood. After blotting excess surface moisture with absorbent paper, the wet weight of the tissues was recorded in grams (g). The lung index (%) was calculated as follows: [lung wet weight (g)/body weight (g)] × 100.

**Table 1:** Experimental design.

Group	*n*	Feed (1–6 days)	Atomization (4–6 days)
N	10	Mouse maintenance fodder	Physiological saline
P	10	Mouse maintenance fodder	LPS solution
G	10	High-calorie fodder	Physiological saline
GP	10	High-calorie fodder	LPS solution

### 2.3 Acetate administration

The treatment of mice in the N and GP groups was the same as described earlier. Additionally, acetate was administered in the drinking water at a concentration of 150 mM (38, 39) for the mice in the GP group.

### 2.4 Drug administration

GLPG-0974 (an inhibitor of GPR43; HY-12940, MedChemExpress) was dissolved in dimethyl sulfoxide and further diluted in 40% PEG300, 5% Tween-80, and 54% saline to a final volume of 10 mL (1%, 0.1 mg/mL). GLPG-0974 was injected intraperitoneally into the GLPG-0974 and GLPG-0974+Acetate groups once daily. Trichostatin A(TSA; an inhibitor of HDAC; HY-15144, MedChemExpress) usage was as described previously, and the mice were given (1.0 mg/kg BW) by i.p (40). The vehicle control group was injected intraperitoneally with equal vehicle (1% dimethyl sulfoxide, 40% PEG300, 5% Tween-80, and 54% saline) at the same time point.

### 2.5 Lung histopathology

Following 24-hour fixation in 4% paraformaldehyde, the lung tissues were dehydrated, paraffin-embedded, and sectioned at a thickness of 5 μm. These sections were then subjected to hematoxylin and eosin (H&E) staining, with subsequent observation and imaging performed under a light microscope (Nikon, Tokyo, Japan). A pathologist blinded to the treatment conducted the histological assessments. The score was graded according to the sum of the score for degree of damage such as hemorrhage, the number of infiltration cells, and edema. Each histological characteristic was assigned a score ranging from 0 to 3 (41).

### 2.6 ELISA

In accordance with the manufacturer’s guidelines, cytokine levels in lung tissue samples were measured using ELISA kits. After homogenization, the samples were centrifuged at 5000 rpm for 5 minutes at 4 °C to obtain the supernatant, which was collected for analysis. Absorbance was determined using a microplate reader (Molecular Devices, Silicon Valley, CA, USA).

### 2.7 Flow cytometric analysis

Lung tissues were harvested on the seventh day. Lung tissues were cut into pieces and incubated in a digestive solution containing type IV collagenase for 0.5 h at 37 °C. After being filtered using a 70-μm cell strainer, cells were centrifuged at 300 ×*g* for 5 min at 4 °C. Then, cell pellets were resuspended at a concentration of 10^7^ cells/mL in 1× PBS buffer. Cell surface Fc receptors were blocked on ice for 10 min using the FcR Blocking Reagent. The cell pellets were stained with antibodies against CD45, CD11b, F4/80, and CD86 in a dark environment at room temperature for 20 min. Cells were then resuspended in 2 mL of PBS with 1% FBS, gently mixed, and centrifuged at 300 × g for 5 min. After discarding the supernatant, the pellet was fixed in 0.5 mL of fixation buffer for 15 min at room temperature in the dark. The cells were then washed twice with 2 mL of 1× permeabilization buffer, centrifuging at 300 × g for 5 min each time. Finally, cells were resuspended in 100 μL permeabilization buffer, stained with 2 μL CD206 antibody for 30 min at room temperature in the dark. Phenotypes of macrophages were identified using the Cytek NL-CLC3000 flow cytometer and analyzed using the FlowJo software.

### 2.8 Fluorescence-activated cell sorting

After lung tissue preparation in a single-cell suspension, cell pellets were resuspended at a concentration of 10^7^ cells/mL. Staining buffer(420201, Biolegend) at a volume of 1 mL was added, and the cells were vortexed, mixed, and centrifuged at 300×g for 5 min. The supernatant was discarded, and cells were enriched. 1μL each of CD45, CD11b, and F4/80 fluorescent- labeled antibodies were added, and the cells were vortexed and incubated in a dark environment for 30 min. Then, they were vortexed and centrifuged at 300×g for 5 min, the supernatant was discarded, and cells were enriched. Staining buffer at a volume of 1 mL was added, and the cells were vortexed and mixed. 7-AAD was added before loading. After detection and sorting, cells were centrifuged, resuspended, and counted. TRIzol was added at −80 °C for subsequent testing.

### 2.9 Magnetic activated cell sorting

After preparation of lung tissue as a single-cell suspension, the cells were vortexed and centrifuged at 300 × g for 10 min. The supernatant was discarded, and the cells were enriched. The cell pellet was resuspended in 90 µL of buffer per 10^7^ total cells. Cells were co-stained with Anti-F4/80 Magnetic Microbeads (130-110-443, Miltenyi Biotec) at a dosage of 10 µL per 10^7^ cells for 15 min at 4°C in the dark. The cells were then washed with 1–2 mL of buffer and centrifuged at 300 × g for 10 min. The supernatant was aspirated, and the cells were resuspended in buffer. The MS column (130-042-201, Miltenyi Biotec) was placed in the magnetic field of a suitable magnetic separator. The column was pre-balanced with 500 µL of buffer, and the cell suspension was applied onto the column. The column was washed with 1.5 mL of buffer. It was then removed from the separator, and the magnetically labeled cells were flushed out into a collection tube with 1 mL of buffer. The magnetically labeled cells were centrifuged at 300 × g for 10 min. The supernatant was aspirated, and the cells were resuspended in PBS for RNA extraction.

### 2.10 Real-time PCR

Total RNA was extracted using TRIzol Reagent according to the manufacturer’s protocol. The RNA concentrations were detected using a spectrophotometer. Reverse transcription was performed using the cDNA Reverse Transcription Kit with 1 ug of RNA, according to the manufacturer’s instructions. Following cDNA synthesis, relative mRNA levels were determined using the CFX96 Real-Time PCR system (Bio-Rad, Hercules, CA, USA) with SYBR Green PCR Master Mix. Data were calculated using the 2^−ΔΔCt^ formula with GAPDH as internal control. The primer sets used are shown in **Table 2**.

**Table 2:** Primer sequences used for RT-PCR analysis.

Gene	Primer	Sequence
*Gpr41*	Forward Reverse	5’- CATGTGGTGGGCTATGTCAG-3’ 5’- CTAGCTCGGACACTCCTTGG -3’
*Gpr43*	Forward Reverse	5’-CTTCCCGGTGCAGTACAAGT-3’ 5’-GCTCTTGGGTGAAGTTCTCG-3’
*Gpr109a*	Forward Reverse	5’- ATGAAAACATCGCCAAGGTC-3’ 5’- CGAACCTCCAGTCCCAGTTA-3’
*Hdac1*	Forward Reverse	5’-TTCCAACATGACCAACCAGA-3’ 5’- ACCACCTTCTCCCTCCTCAT-3’
*Hdac2*	Forward Reverse	5’-ACCCGGACAAAAGAATTTCC-3’ 5’-TTGGGGTCTGTTTTCTCACC -3’
*Hdac3*	Forward Reverse	5’-AATGTGCCCTTACGAGATGG-3’ 5’-GTAGCCACCACCTCCCAGTA-3’
*Hdac4*	Forward Reverse	5’- CCAAGGTTCACCACAGGTCT-3’ 5’- TTGTGCCGTAGAGGAGTGTG-3’
*Hdac5*	Forward Reverse	5’-AGTGAGAGCACCCAGGAAGA-3’ 5’- GTACACCTGGAGGGGCTGTA-3’
*Hdac6*	Forward Reverse	5’-CTGGCTAAGGGAGTCAGTGC-3’ 5’-TAGCACGGCTTCTTCCACTT-3’
*Hdac7*	Forward Reverse	5’-TGGAGACAACAGCAAGCATC-3’ 5’- TCCCGTTATCCAGTTTGAGG-3’
*Hdac8*	Forward Reverse	5’- TGCCCTGCATAAACAAATGA-3’ 5’- GGCTGGGCAGTCATAACCTA-3’
*Hdac9*	Forward Reverse	5’- CTCAGAGCCCAACTTGAAGG-3’ 5’- GCCTCATTTTCGGTCACATT-3’
*Hdac10*	Forward Reverse	5’- GCCTCATTCTGGGTCTGGTA-3’ 5’- GCGTATGGATTCCTCTTCCA-3’
*Hdac11*	Forward Reverse	5’- AGTGAGAGCACCCAGGAAGA-3’ 5’- GTACACCTGGAGGGGCTGTA-3’
*Hif-1α*	Forward Reverse	5’- GCGGCCCGGAGTCTAAAGTA-3’ 5’- GGGGCCATCCACAGTCTTCT-3’
*Gapdh*	Forward Reverse	5’-ATGGGTGTGAACCACGAGA-3’ 5’-CAGGGATGATGTTCTGGGCA-3’

### 2.11 Fecal/serum/lung tissue SCFAs analysis

Fecal samples weighing 30 mg was diluted in 50 μl of 20% phosphoric acid, and centrifuged at 14,000×g for 20 min. In the upper organic phase of 600 μl,4-methylvaleric acid with a final concentration of 500 μM was added as an internal standard, and then 1 μl supernatant was absorbed and detected by GC–MS with an Agilent DB-WAX capillary GC column with a split ratio of 10:1. The initial temperature was maintained at 90°C, and then raised to 160°C at a rate of 10°C/min. After that, the temperature was raised to 150°C at a rate of 5°C/min and maintained at 150°C for 2 min. Finally, the temperature was raised to 240°C at a rate of 40°C/min and maintained at 240°C for 5 min. Helium was used as carrier gas. An Agilent 5977B MSD mass spectrometer was used for mass spectrometry analysis. The MSD ChemStation software was used to extract the chromatographic peak area and retention time. A standard curve was drawn, and the content of SCFAs in the sample was calculated. The serum sample volume was 100 μL, and the lung tissue sample weight was 30 mg. The remaining assay procedures were identical to those used for the fecal SCFA analysis.

### 2.12 Acetate quantification

Serum samples can be assayed directly using the Acetate Colorimetric Assay Kit(MAK086, Sigma)according to the manufacturer’s protocol. Lung tissue (10 mg) was homogenized in 100 μL of ice-cold Acetate Assay Buffer. Following centrifugation at 13,000 × g for 10 min, the precipitate was discarded, and the supernatant was collected and assayed using the identical protocol described for serum samples.

### 2.13 Immunofluorescence

For staining lung tissues, thin sections were blocked with 1% bovine serum albumin, followed by incubation with primary antibodies against HIF-1α (dilution, 1:100), F4/80 (dilution, 1:100) and GFP (dilution, 1:100) at 4°C overnight. After three times washing with PBS, the sections on slides were incubated with fluorescence-conjugated secondary antibodies (dilution, 1:100) at room temperature for 2 h. Sections incubated with secondary antibodies alone were used as negative controls. Finally, DAPI was used to stain the nuclei of cells. Images were taken at random fields under a Eclipse Fi3 microscope (Nikon, Japan).

### 2.14 Mice transfected with adeno-associated virus–*Hdac9/10*

An adeno-associated virus (AAV) vector expressing *H* ***dac*** *9* and *H* ***dac*** *10* were obtained from Genomeditech Co. Ltd.AAV at a dose of 2×10^11^ vg in 40 µL of PBS per mouse was administered to mice through intratracheal injection. The transfection efficiency was confirmed by Immunofluorescence and PCR.

### 2.15 HDAC activity

HDAC activity analysis was performed using the HDAC activity fluorometric assay Kit(E-BC-F051,Elabscience). Absorbance was determined using a microplate reader (Molecular Devices, Silicon Valley, CA, USA).

### 2.16 Statistical analysis

Data were analyzed using GraphPad Prism 8 software(La Jolla, CA, USA) and expressed as mean ± SD, and are representative of at least 3 independent experiments. The differences among different groups were compared by One-way ANOVA analysis. *P* < 0.05 was considered statistically significant.

## 3 Results

### 3.1 A high-calorie diet significantly aggravates LPS-induced pneumonia in juvenile mice

**Figure 1:**
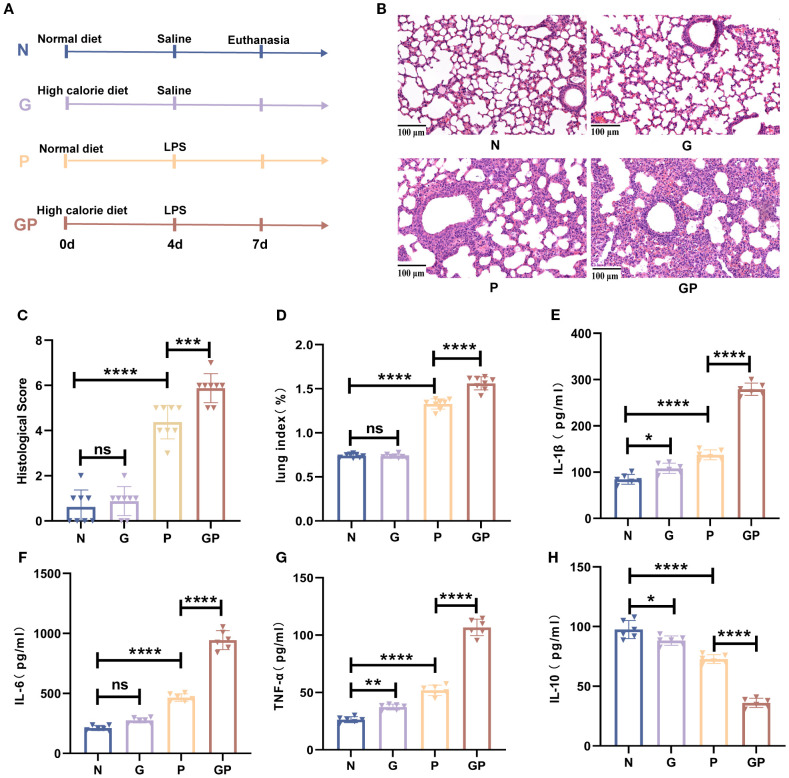
A high-calorie diet significantly aggravates LPS-induced pneumonia in juvenile mice. **(A)** The experimental design. **(B)** H&E staining (100×). **(C)** Quantified scoring of pulmonary pathology. **(D)** The lung index of mice. **(E–H)** IL-1β, IL-6,IL-10 and TNF-α levels in the lung tissue were quantified using ELISA. Data are represented as mean ± SD (*n* = 6-8). *p < 0.05, **p < 0.01, ***p < 0.001, ****p < 0.0001, and ns (*p* > 0.05).

The influence of a high-calorie diet on LPS-induced pneumonia was investigated. A high-calorie diet was administered orally daily for 3 days before LPS nebulization, and mouse maintenance fodder was used as a control (**Figure 1A**). The severity of lung inflammation injury was further evaluated by histopathologic analysis. After LPS nebulization, alveolar wall thickening, alveolar and interstitial inflammatory cell infiltration, alveolar exudate, and edema were observed in the lung tissues of mice in the P group. The alveolar structure of the mice in the GP group fed a high-calorie diet was unclear, fused, or even disappeared and accompanied by a high degree of inflammatory cell infiltration (**Figures 1B, C**). A high-calorie diet also increased the lung index in mice with LPS-induced pneumonia compared with that in the P group (**Figure 1D**).

To understand the proinflammatory effect of a high-calorie diet on LPS-induced pneumonia, we measured the levels of proinflammatory cytokines in the lung tissue. The results showed that the levels of proinflammatory cytokines, such as IL-1β, TNF-α, and IL-6, were significantly increased after LPS challenge in the lung tissues and that anti-inflammatory cytokine IL-10 was decreased (**Figures 1E–H**). Compared with those in the P group, these proinflammatory cytokines were further increased significantly, and anti-inflammatory cytokine was decreased in the GP group (**Figures 1E–H**). Taken together, our results indicate that a high-calorie diet could aggravate pulmonary inflammatory injury induced by LPS.

### 3.2 A high-calorie diet aggravates M1/M2 imbalance in juvenile mice with LPS-induced pneumonia

**Figure 2:**
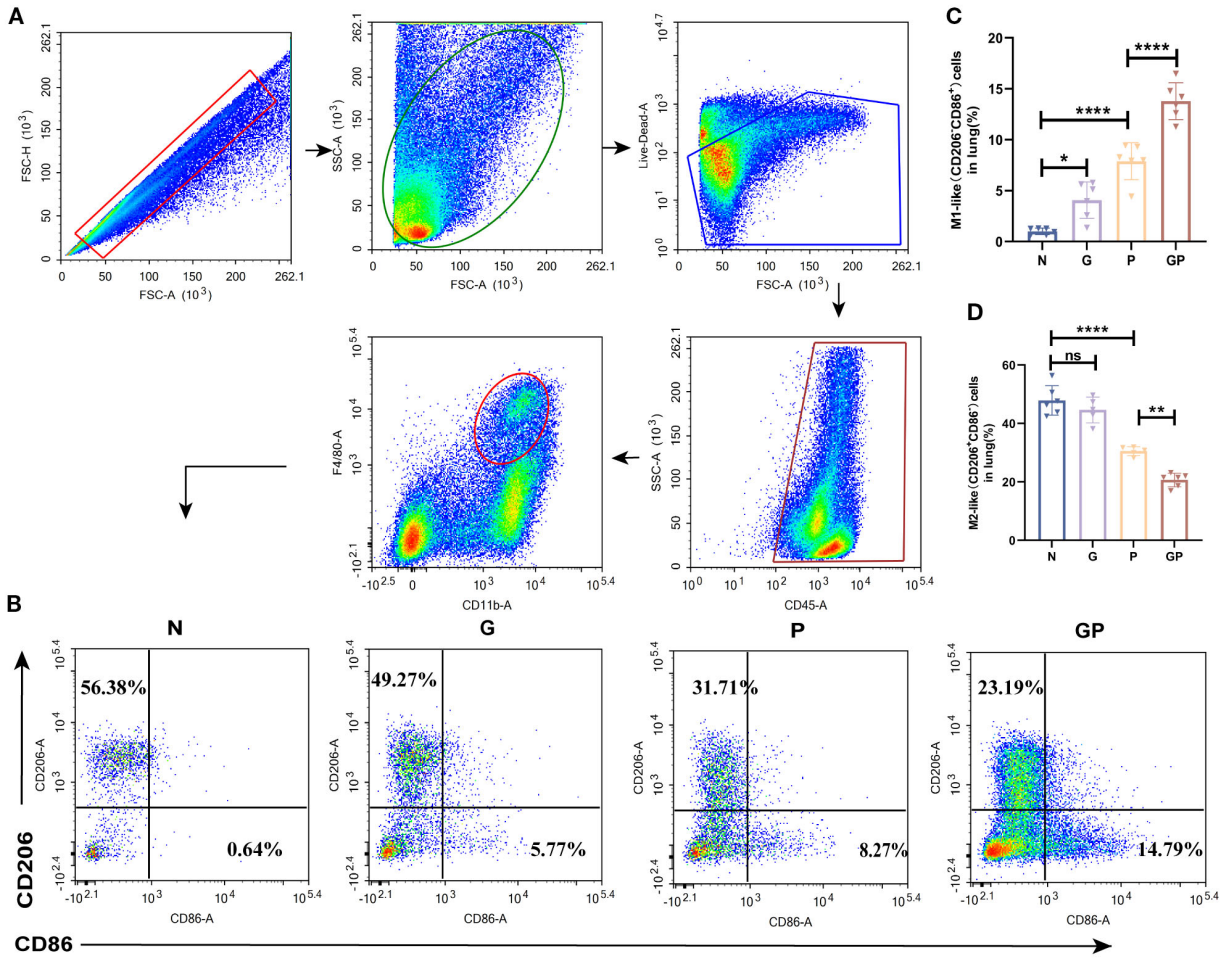
A high-calorie diet exacerbates the macrophage polarization imbalance. **(A)** The flow cytometry gating strategy. **(B–D)** The proportions of M1-like(CD206-CD86+) and M2-like (CD206+ CD86-) macrophages in lung tissue were detected by flow cytometry. Data are represented as mean ± SD (n = 5-6). *p < 0.05, **p < 0.01, ****p < 0.0001, and ns (*p* > 0.05).

Given the central role of macrophages as the first line of defense against pathogens and their polarization imbalance in driving severe pulmonary inflammation and acute lung injury, we investigated how a high-calorie diet influences lung macrophage polarization in a mouse model of pneumonia. We found that a high-calorie diet significantly increased the proportion of M1-like (CD206^−^CD86^+^) macrophages in lung tissue (*P* < 0.05), while showing a trend toward reducing the proportion of M2-like (CD206^+^CD86^−^) macrophages, though this did not reach statistical significance. After LPS nebulization, the GP group exhibited a marked increase in M1-like (CD206^−^CD86^+^) macrophages and a significant decrease in M2-like (CD206^+^CD86^−^) macrophages in lung tissue compared to the P group, resulting in a pronounced M1/M2 polarization imbalance (**Figures 2A–C**). This showed that a high-calorie diet aggravated lung macrophage polarization in pneumonia model mice.

### 3.3 A high-calorie diet decreases concentrations of SCFAs

**Figure 3:**
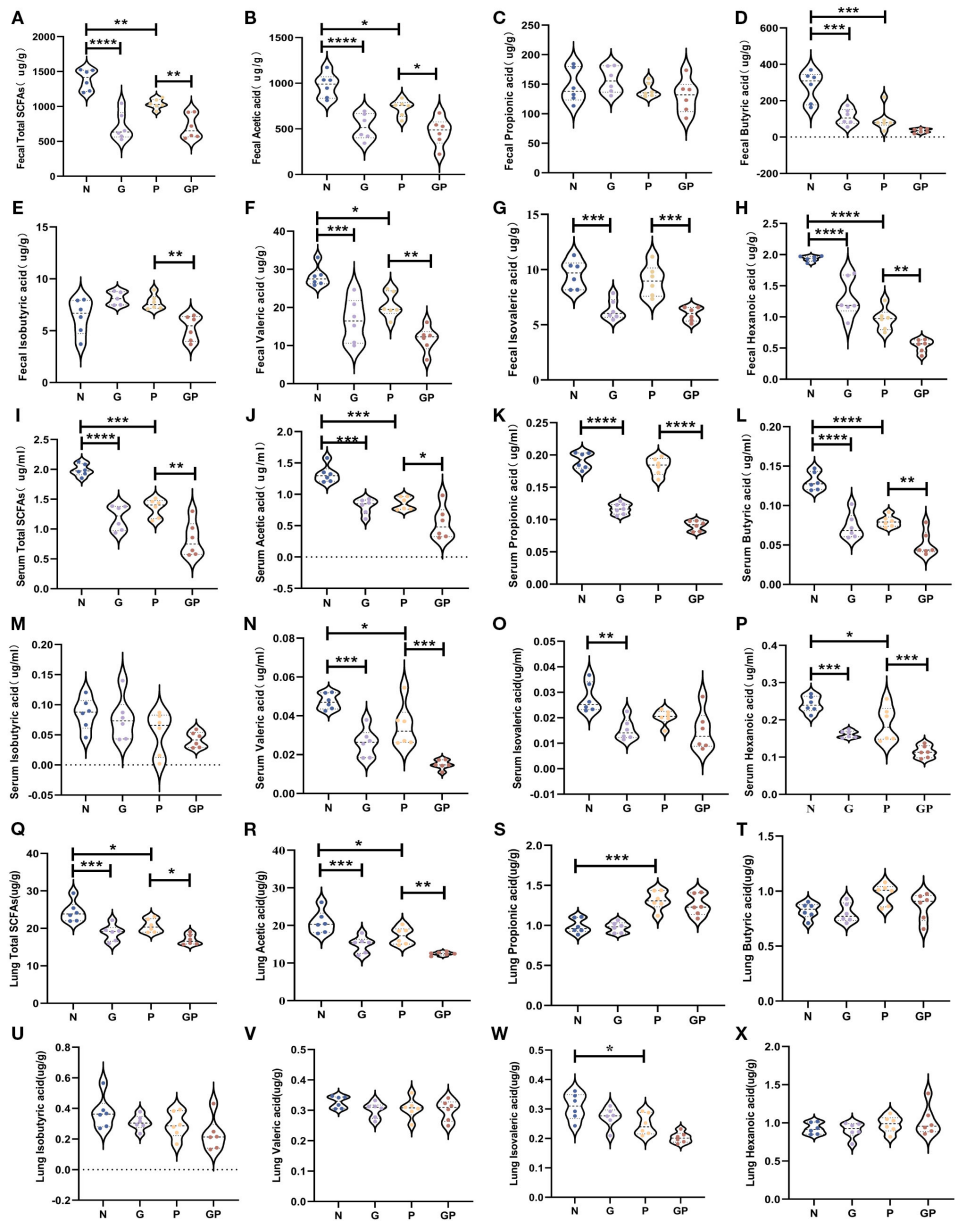
A high-calorie diet decreases the level of SCFAs in feces、serum and lung tissue.**(A)** Total SCFAs concentrations in feces. **(B–H)** Acetic, propionic, butyric, isobutyric, valeric, isovaleric, and hexanoic acid concentrations in feces. **(I)** Total SCFAs concentrations in Serum. **(J–P)** Acetic, propionic, butyric, isobutyric, valeric, isovaleric, and hexanoic acid concentrations in serum. **(Q)** Total SCFAs concentrations in lung tissue. **(R–X)** Acetic, propionic, butyric, isobutyric, valeric, isovaleric, and hexanoic acid concentrations in serum. Data are represented as mean ± SD (n = 6). *p < 0.05,**p < 0.01, ***p < 0.001, ****p < 0.0001.

Studies have found that a high-fat and high-calorie diet damages the gut barrier, changes the gut microbiota structure, and decreases SCFA production (13, 42). Furthermore, increasing evidence shows that SCFAs participate in the host defense against respiratory tract infection (22) and affect the severity of acute respiratory tract disease (23). Therefore, we used targeted metabolomics to measure levels of SCFAs in stool. SCFAs showed varying levels of decreases in the G and GP groups, particularly acetic acid, valeric acid, isovaleric acid, and hexanoic acid (**Figures 3A–H**). It is well known that gut SCFAs can reach the distal organs via systemic circulation to carry out their immunomodulation effects. Therefore, we also measured SCFA levels in blood and lung tissues. Serum SCFAs showed varying levels of decreases in the G and GP groups, particularly acetic acid, propionic acid, butyric acid, valeric acid, and caproic acid (**Figures 3I–P**); the decrease in lung tissue SCFA levels was mainly owing to acetic acid (**Figure 3Q–X**). In summary, a high-calorie diet decreased SCFA levels in model mouse stool, serum, and lung tissues, with acetic acid mainly decreased in lung tissues.

### 3.4 Mechanisms by which a high-calorie diet aggravates pneumonia are associated with the resultant decline in acetate levels

**Figure 4:**
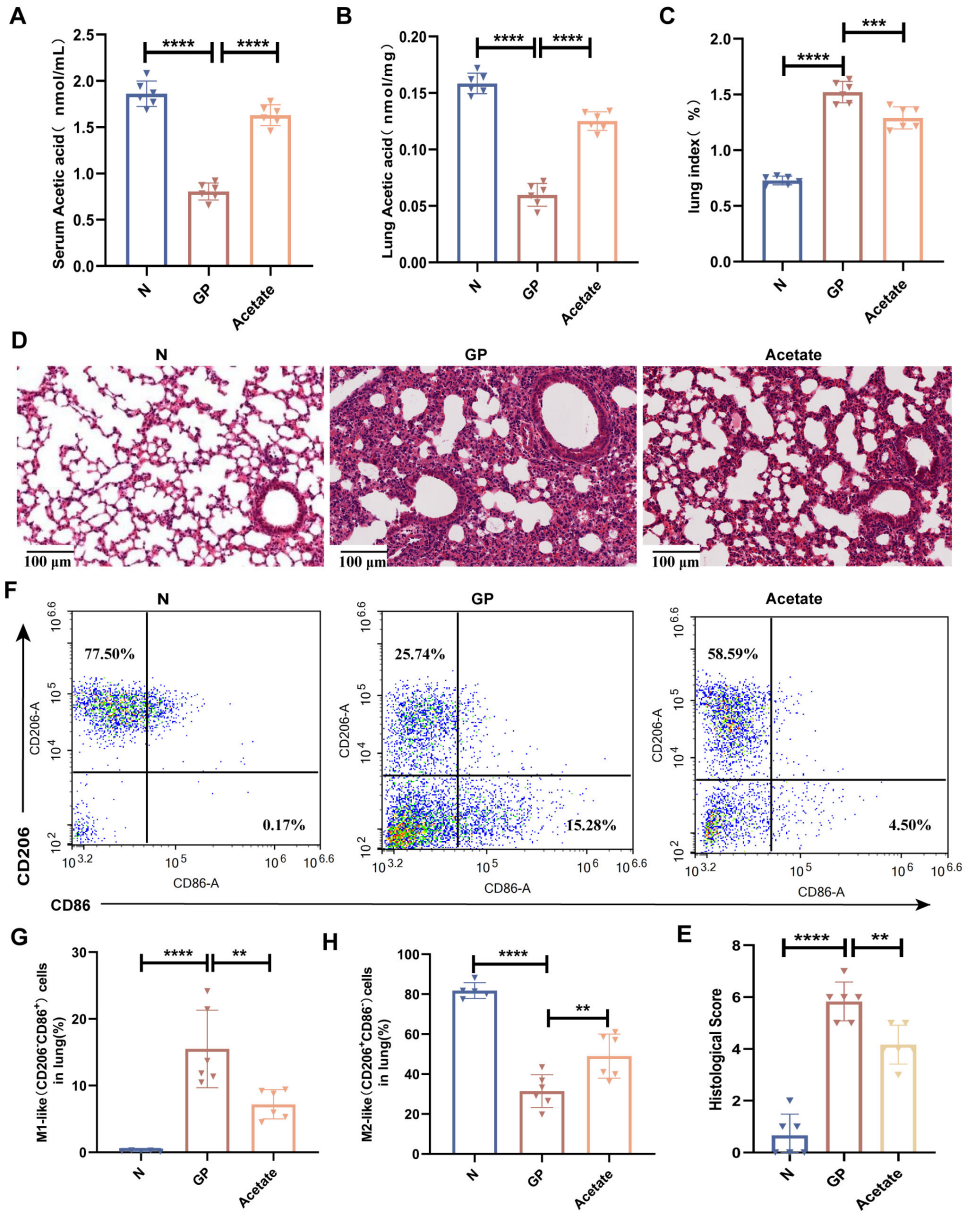
Mechanisms by which a high-calorie diet aggravates pneumonia are associated with the resultant decline in acetate levels. **(A)** Acetic concentrations in serum. **(B)** Acetic concentrations in lung tissue. **(C)** The lung index of mice. **(D)** H&E staining (100×). **(E)** Quantified scoring of pulmonary pathology. **(F–H)** The proportions of M1-like(CD206^-^CD86^+^) and M2-like (CD206^+^ CD86^-^) macrophages in lung tissue were detected by flow cytometry. Data are represented as mean ± SD (*n* = 5- 6). ^**^ *p* < 0.01, ^****^ *p* < 0.0001.

SCFAs are extremely important for the maintenance of lung homeostasis, and lung SCFA levels determine the baseline lung immunity levels at rest and may be vital for immune responses in lung injury (20). One study showed that gut microorganism-derived acetate can increase the host defense against respiratory syncytial virus and influenza A virus (43, 44). Our study also found that a high-calorie diet induced a significant decline in acetate levels in mouse stool, serum, and lung tissues. We wished to determine whether the mechanism via which a high-calorie diet aggravates pneumonia is associated with high-calorie diet-mediated decrease in acetate. Therefore, an SCFA refeeding experiment was used for validation during the experimental period. In model construction, drinking water was used for acetate supplementation in mice. We found that acetate levels in serum and lung tissue were significantly increased (**Figures 4A, B**) in mice. After acetate supplementation, the mouse lung index was significantly decreased (**Figure 4C**) and the pulmonary inflammatory injury was decreased (**Figures 4D, E**). We also found that the proportion of M1 macrophages was decreased, the proportion of M2 macrophages was increased, and the M1/M2 polarization imbalance was decreased in mouse lung tissues after acetate supplementation, as compared with the GP group (**Figures 4F-H**, **Supplementary Figure S1**). These data show that the mechanism via which a high-calorie diet aggravates pneumonia is associated with the resultant acetate decrease, and acetate supplementation could alleviate M1-like(CD206^-^CD86^+^)/M2-like(CD206^+^CD86^-^) macrophage imbalance in GP group mice as well as lung inflammatory injury in mice.

### 3.5 Acetate reduction caused by a high-calorie diet mainly affects HDAC 9 and HDAC10 in lung macrophages

**Figure 5:**
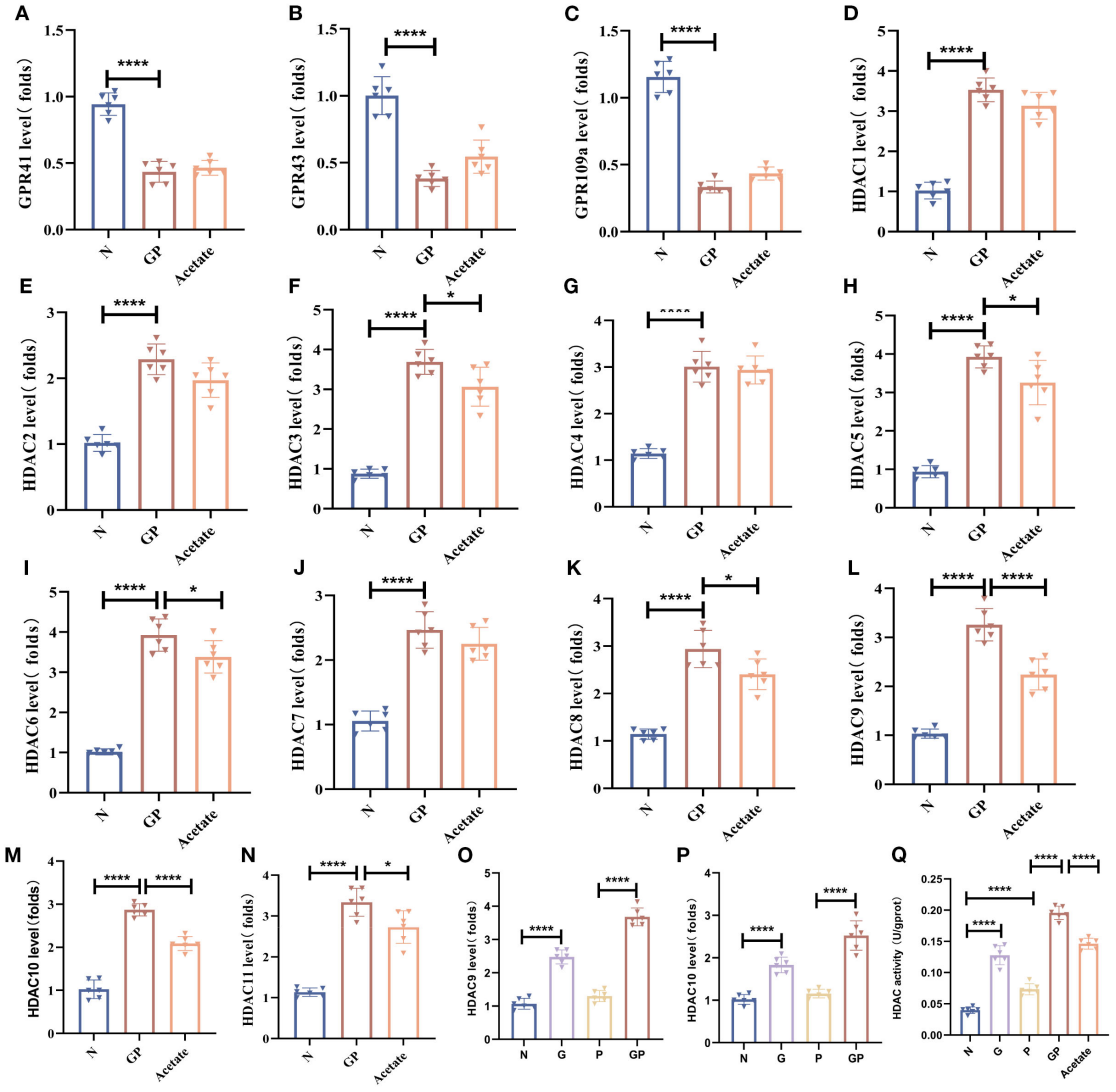
Acetate reduction caused by a high-calorie diet mainly affects HDAC9 and HDAC10 in lung macrophages. **(A–C)** The mRNA level of *GprR41/Gpr43/Gpr109a* in the lung macrophage. **(D–P)** The mRNA level of *Hdac(1-11)* in the lung macrophage. **(Q)** HDAC activity in the lung macrophage. Data are represented as mean ± SD (*n* = 6). ^*^ *p* < 0.05, ^****^ *p* < 0.0001.

Our study found that acetate could drive changes in the lung macrophage phenotype whereas acetate mainly carries out its effects via GPCR activation and HDAC inhibition (24, 25). To investigate how a high-calorie diet-induced acetate decrease affects lung macrophage phenotypic changes, we isolated lung macrophages by flow cytometry and measured major GPCRs (GPR41, GPR43, GPR109A) and all canonical HDACs (HDAC1–11). We found that *Gpr43* mRNA levels were increased in lung tissue macrophages in GP group mice after acetate supplementation (**Figure 5B**); however, this was not statistically significant. There was no significant change in *Gpr41* and *Gpr109a* mRNA levels (**Figures 5A, C**), and *Hdacs 1–11* were downregulated, among which *Hdac9* and *Hdac10* showed significant differences (**Figures 5D-N**). This shows that acetate supplementation could significantly decrease *Hdac9* and *Hdac10* expression levels in lung tissue macrophages in GP mice. We also found that a high-calorie diet increases *Hdac9* and *Hdac10* expression levels in lung tissue macrophages, whereas LPS nebulization alone did not alter the mRNA levels of *Hdac9* or *Hdac10* (**Figures 5O, P**), indicating that a high-calorie diet is the primary factor responsible for these changes. We also measured HDAC activity levels, and the results showed that a high-calorie diet increased HDAC activity, while acetate supplementation reduced HDAC activity (**Figure 5Q**). Therefore, our study results showed that a decrease in acetate induced by a high-calorie diet may affect lung macrophage *Hdacs*, which was most pronounced for *Hdac9* and *Hdac10*. However, whether the mechanisms via which a high-calorie diet-induced acetate decrease drives lung tissue macrophage phenotypic changes is associated with *Hdac9* and *Hdac10* requires further study.

### 3.6 Acetate’s regulation of lung tissue M1/M2 balance and alleviation of inflammatory injury are associated with HDAC rather than GPR43

**Figure 6:**
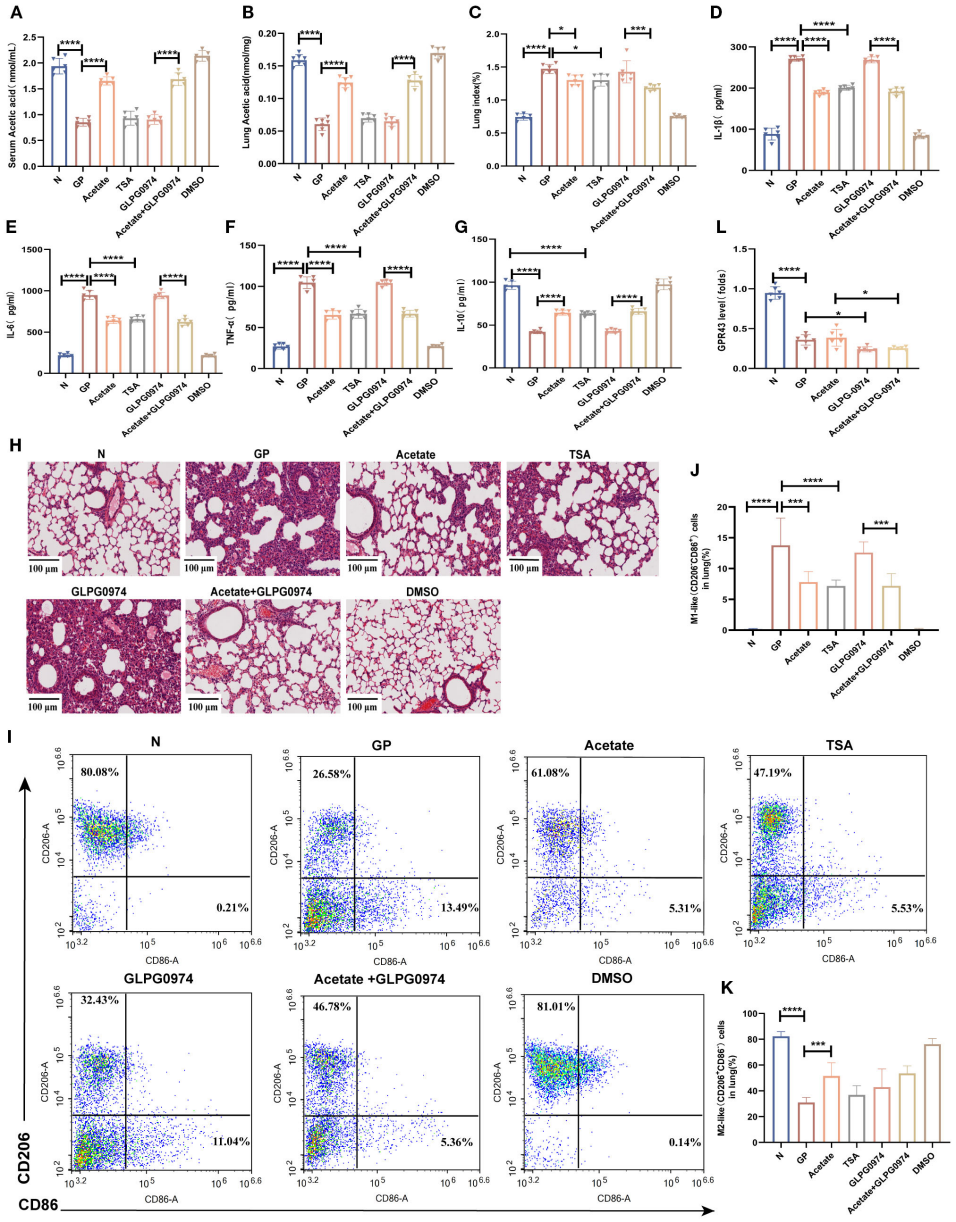
Acetate’s regulation of lung tissue M1/M2 balance and alleviation of inflammatory injury are associated with HDAC rather than GPR43. **(A)** Acetic concentrations in serum. **(B)** Acetic concentrations in lung tissue. **(C)** The lung index of mice. **(D–G)** IL-1β, IL-6,IL-10 and TNF-α levels in the lung tissue were quantified using ELISA. **(H)** H&E staining (100×). **(I–K)** The proportions of M1-like (CD206^-^CD86^+^)/M2-like(CD206^+^CD86^-^) in lung tissue were detected by flow cytometry. **(L)** The mRNA level of *Gpr43* in the lung macrophage. Data are represented as mean ± SD (*n* = 6). ^*^ *p* < 0.05, ^***^ *p* < 0.001, ^****^ *p* < 0.0001.

To further verify whether the mechanism via which a high-calorie diet regulates the lung macrophage M1/M2 balance through HDAC and not GPR43, we used the pan-HDAC inhibitor TSA (**Supplementary Table S2**). We supplemented acetate via drinking water without affecting food intake (**Supplementary Figure S2**), and the results showed that acetate levels were increased in both lung tissue and serum (**Figures 6A, B**). The results showed that both acetate and TSA similarly decreased the proportion of M1-like (CD206^-^CD86^+^) macrophages and restores the M1-like(CD206^-^CD86^+^)/M2-like(CD206^+^CD86^-^) polarization balance (**Figures 6I–K**). Additionally, both acetate and TSA reduced the expression of pro-inflammatory cytokines such as TNF-α, IL-1β, and IL-6 in lung tissues (**Figures 6D–F**), while elevating the anti-inflammatory cytokine IL-10 (**Figure 6G**). Furthermore, these treatments lowered the lung index (**Figure 6C**) and attenuated inflammatory injury in lung tissue (**Figure 6H**). We also used a GPR43 inhibitor to further validate whether acetate is dependent on GPR43 to carry out its effects. We measured the mRNA expression level of *Gpr43* in pulmonary macrophages. The results showed that compared with the GP group, the mRNA level of *Gpr43* decreased after treatment with the GPR43 inhibitor (**Figure 6L**). But the results showed that addition of a GPR43 inhibitor on top of a high-calorie diet did not significantly affect lung inflammatory injury caused by subsequent LPS nebulization. And there were no significant differences in the lung index (**Figure 6C**), lung cytokines (**Figures 6D–G**), or lung histopathology (**Figure 6H**) compared with the GP group. After addition of a GPR43 inhibitor, acetate supplementation in the GP group still similarly decreased the proportion of M1 macrophages (**Figures 6I, J**), and there were no significant difference in the lung index, lung tissue inflammatory factors, and degree of lung inflammatory injury, as compared with the non-inhibitor group. These results suggest that a high-calorie diet is associated with macrophage polarization imbalance in lung tissue, which may be linked to a diet-induced reduction in acetate and related HDAC inhibition, but not to GPR43.

### 3.7 Acetate’s reduction of macrophage HIF-1α expression in GP group mice may be associated with HDAC inhibition

**Figure 7:**
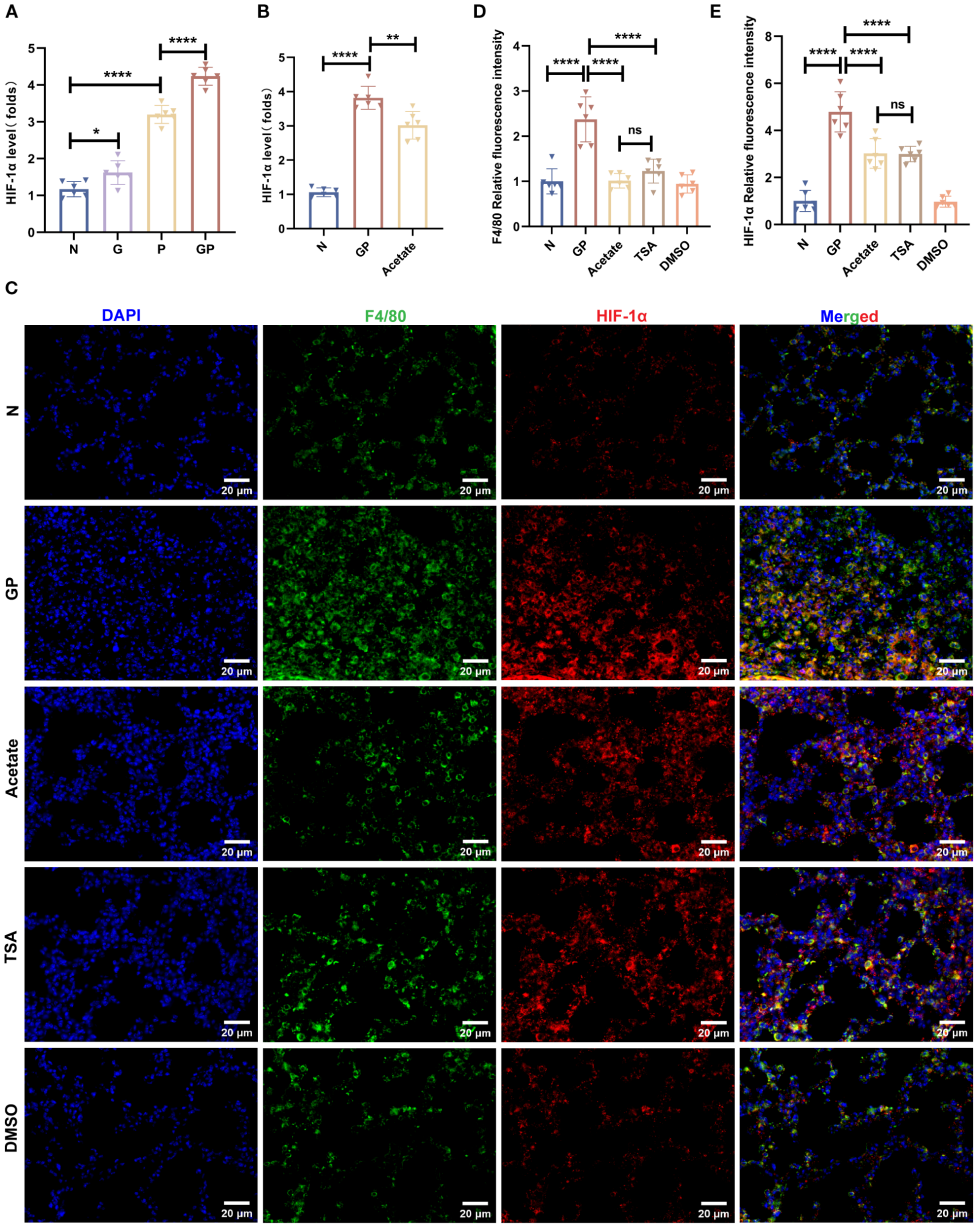
Acetate’s reduction of macrophage HIF-1a expression in GP group mice may be associated with HDAC inhibition. **(A, B)** The mRNA level of HIF-1a in the lung macrophage. **(C–E)** Representative images of co-immunofluorescent staining of HIF-1a with F4/80 in lungs. Blue: DAPI; Green: F4/80; Red: HIF-1a. Data are represented as mean± SD (*n* = 6). *p < 0.05, **p < 0.01, ****p < 0.0001, and ns (*p* > 0.05).

HIF-1α activation can promote macrophage glycolysis and is associated with polarization toward the M1 pro-inflammatory macrophage phenotype (36). To investigate the mechanism by which acetate drives phenotypic changes in macrophages, We used flow cytometry to sort macrophages from lung tissues and measured HIF-1α transcript levels in macrophages. The results showed that HIF-1α transcript levels were significantly increased in macrophages from pneumonia model mice and those fed a high-calorie diet had even higher HIF-1α transcript levels (**Figure 7A**). This shows that a high-calorie diet increases HIF-1α expression in pneumonia model mouse macrophages, which is consistent with an increase in the proportion of M1-like(CD206^-^CD86^+^) macrophages. After acetate supplementation, HIF-1α expression levels in lung macrophages from GP group mice were decreased (**Figure 7B**), showing that the increase of macrophage HIF-1α expression levels in pneumonia model mice caused by a high-calorie diet may be associated with the resulting decrease in acetate level. Interestingly, the HDAC inhibitor TSA also decreased HIF-1α expression in lung tissue macrophages (**Figure 7C**). This finding is consistent with the possibility that acetate reduces HIF-1α levels in GP group mice through a mechanism that may involve HDAC inhibition.

### 3.8 Acetate interferes with lung tissue macrophage M1/M2 polarization, which is associated with the HDAC9/10/HIF-1α/glycolysis pathway

**Figure 8:**
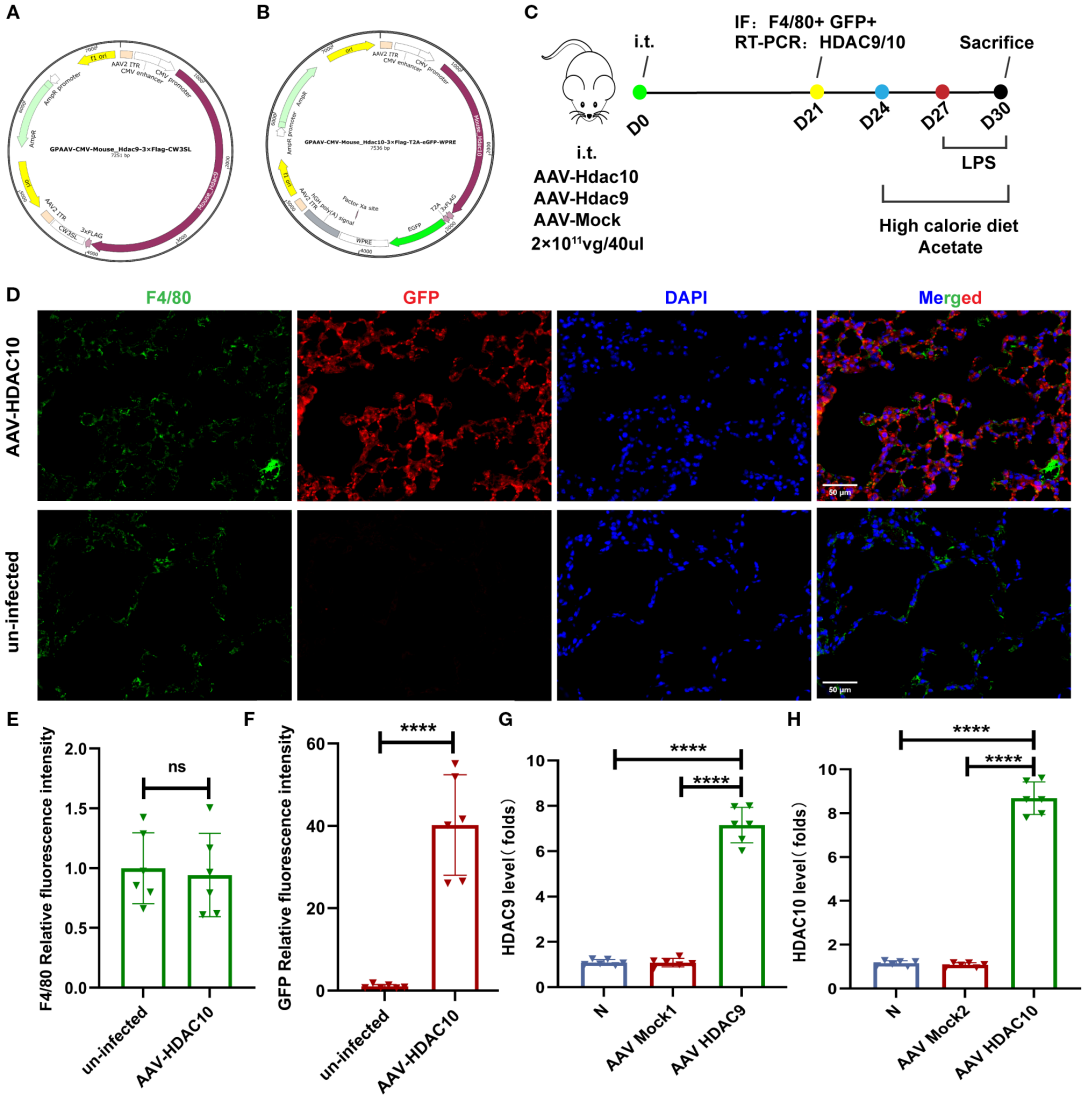
Mouse model construction for overexpressing *Hdac9* and *Hdac10*. **(A)** Full sequence map for GPAAV-CMV-*Hdac9*-3×Flag-CW3SL. **(B)** Full sequence map for GPAAV-CMV-*Hdac10*-3×Flag-T2A-eGFP-WPRE. **(C)** The experimental design. **(D–F)** Representative images of co-immunofluorescent staining of GFP with F4/80 in lungs. Blue: DAPI; Green: F4/80; Red: GFP. **(G, H)** The mRNA level of Hdac9 and Hdac10 in the lung macrophage. Data are represented as mean ± SD (*n* = 6). ****p < 0.0001, and ns (*p* > 0.05).

Our earlier data showed that a high-calorie diet could decrease acetate, which was associated to with attenuated HDAC inhibition and accompanied by increased HIF-1α expression in macrophages from pneumonia model mice. After acetate intervention, changes in *Hdac9* and *Hdac10* were the most significant. Therefore, to further clarify the main HDAC subtypes involved in the high-calorie diet-mediated acetate decrease and examine the mechanisms by which this affects macrophage phenotypes, we used adeno-associated viral vector endotracheal intubation and infusion for construction of mice overexpressing *Hdac9* and *Hdac10* (**Figures 8A, B**). On day 21, immunofluorescence was used to measure GFP levels to evaluate the transfection results of the AAV *Hdac10* vector (**Figure 8C**). The results showed that F4/80 and GFP colocalization were significantly increased in the lung tissue of mice transfected with AAV *Hdac10*, compared with the non-transfected group (**Figures 8D-F**). We used magnetic beads to obtain macrophages and PCR to measure *Hdac10* transcript levels. The results showed that transcript levels were significantly increased (**Figure 8H**), indicating that the AAV *Hdac10* plasmid vector had been transfected into macrophages. Because the *Hdac9* gene sequence is very long, a fluorescence tag was not added for the plasmid vector. We measured *Hdac9* mRNA expression levels in lung macrophages; the results showed that the transcript level was significantly increased (**Figure 8G**). These data showed that *Hdac9*- and *Hdac10*-overexpressing mouse models were successfully constructed.

**Figure 9:**
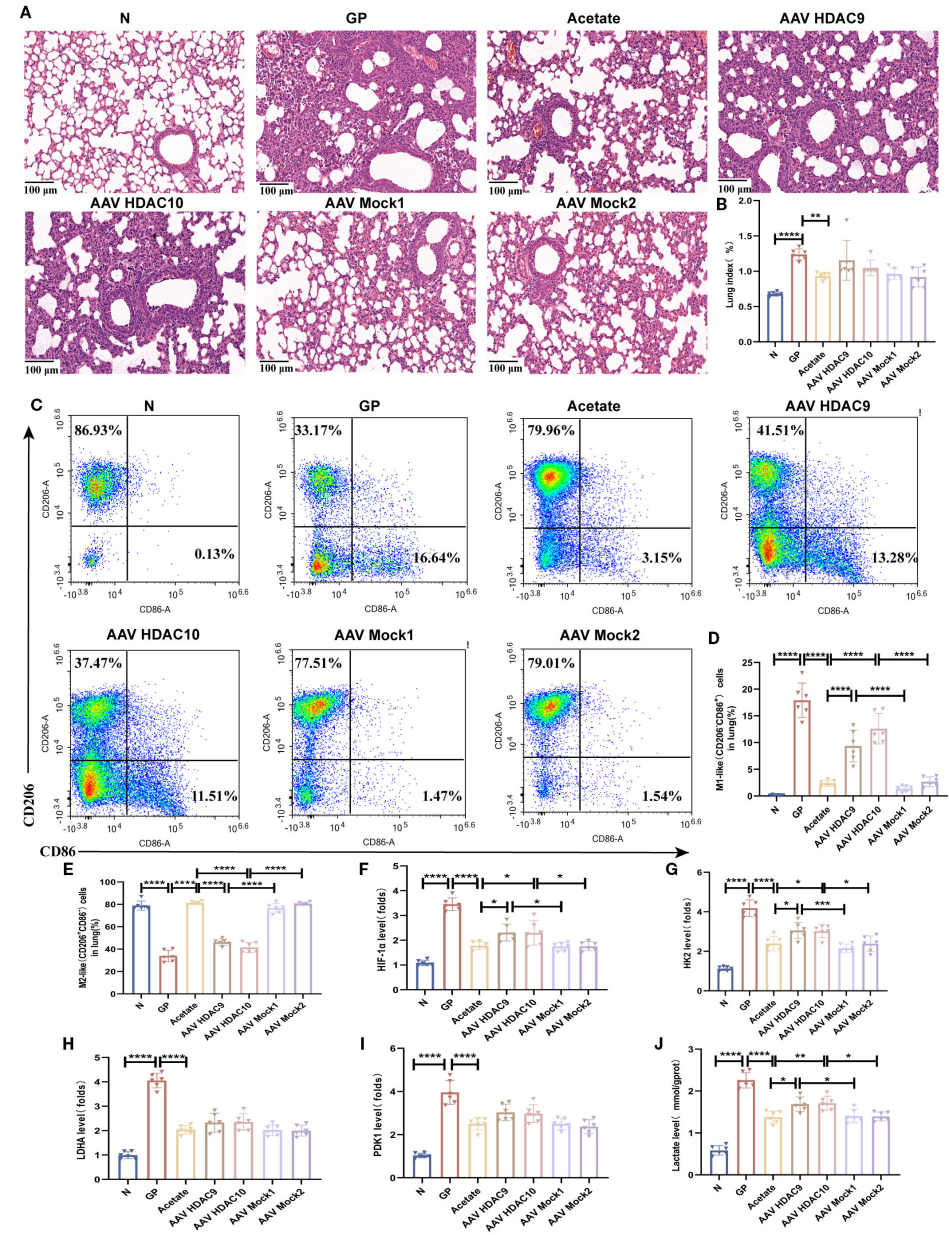
Acetate interferes with lung tissue macrophage M1/M2 polarization, which is associated with the HDAC9/10/HIF-1α/glycolysis pathway. **(A)** H&E staining (100×). **(B)** The lung index of mice. **(C–E)** The proportions of M1-like (CD206^-^CD86^+^)/M2-like(CD206^+^CD86^-^) in lung tissue were detected by flow cytometry. **(F–I)** The mRNA level of *Hif-1α, Hk2, Ldha* and *Pdk1* in the lung macrophage. **(J)** The concentration of lactate in the lung macrophage. Data are represented as mean ± SD (*n* = 6). ^*^ *p* < 0.05,^**^ *p* < 0.01, ^***^ *p* < 0.001, ^****^ *p* < 0.0001.

Related experimental model construction was carried out on this basis (**Figure 8C**, **Supplementary Figure S3**). The results showed that compared with the acetate group, lung tissue inflammatory injury (**Figure 9A**) and lung tissue macrophage M1-like(CD206^-^CD86^+^)/M2-like(CD206^+^CD86^-^) polarization imbalance was increased after *Hdac9* and *Hdac10* overexpression (**Figures 9C–E**, **Supplementary Figure S3**). This result suggests that acetate’s mediating effect in high-calorie diet-aggravated pneumonia may involve *Hdac9/Hdac10*. The question arises of how acetate intervention of *Hdac9/10* further drives macrophage phenotypic changes. We found that acetate decreased lung tissue macrophage HIF-1α (**Figure 9F**), HK2 (**Figure 9G**), LDHA (**Figure 9H**), and PDK1 (**Figure 9I**) transcript levels and lactic acid levels (**Figure 9J**) in pneumonia mouse models fed a high-calorie diet. This demonstrates that acetate decreases glycolysis levels in lung tissue macrophages from pneumonia model mice fed a high-calorie diet and that glycolysis can drive M1-like(CD206^-^CD86^+^) macrophage polarization, which was consistent with macrophage phenotypic changes detected in flow cytometry. After *Hdac9* and *Hdac10* overexpression, the effects of acetate on decreasing lung tissue macrophage HIF-1α expression (**Figure 9F**) and lactic acid levels (**Figure 9J**) in pneumonia model mice fed a high-calorie diet were attenuated, which was consistent with the results of flow cytometry. However, we found that after *Hdac9* and *Hdac10* overexpression, the effects of acetate in decreasing lung tissue macrophage *Hk2* mRNA levels in pneumonia model mice fed a high-calorie diet were attenuated (**Figure 9G**) but this had no significant effect on *Ldha* and *Pdk1* transcript levels (**Figures 9H, I**). This finding indicates that *Hk2* may be a potential downstream target through which acetate, potentially via *Hdac9/10*, influences glycolysis. The activation of HIF-1α upregulates glycolysis in macrophages, a process linked to the pro-inflammatory M1 phenotype (36). Therefore, these results suggest that acetate is associated with macrophage phenotypic changes, potentially involving the HDAC9/10–HIF-1α–glycolysis pathway.

## 4 Discussion

Evidence shows that the gut microbiota can affect lung immunity through multiple mechanisms (45). As a main microflora metabolite that regulates the gut-lung axis (46), SCFAs are extremely important maintaining lung homeostasis and regulating the defense mechanisms of alveolar cells (47). Decreased SCFA levels will affect lung immunity (48). However, the specific mechanisms by which SCFAs interfere with lung immunity to affect pneumonia occurrence and progression are still unclear. In the present study, we proved that a high-calorie diet can decrease gut microbiota metabolite SCFA levels to promote macrophage polarization imbalance, thereby aggravating lung inflammatory injury. Our data suggest that the acetate reduction induced by a high-calorie diet was associated with a sequence of events involving HDAC9/10, HIF-1α, and glycolysis, which may collectively influence macrophage polarization. Our study results demonstrate the important effects of the gut microbiota metabolite acetate in pneumonia progression and suggests a potential strategy in which acetate can be used for intervention against infectious and inflammatory diseases.

A high-calorie diet has long been linked to chronic inflammatory diseases (49), with most studies focusing on its effects over three weeks to several months, primarily in the context of systemic inflammation and metabolic disorders. Recent evidence suggests that even short-term high-calorie intake may temporarily suppress mucosal and systemic immunity, increasing susceptibility to pathogens (50). Our study shows that just six days of a high-calorie diet elevated pro-inflammatory cytokines (IL-1β and TNF-α) in lung tissue without causing full pulmonary inflammation. It also increased the proportion of M1-like(CD206-CD86+) macrophages without affecting M2-like(CD206+CD86-) macrophages, indicating a disruption in pulmonary immune balance. When mice on this diet were exposed to LPS nebulization, they developed more severe lung inflammation, with higher pro-inflammatory cytokines, lower anti-inflammatory cytokines, increased M1-like(CD206-CD86+) macrophages, and decreased M2-like(CD206+CD86-) macrophages. These results suggest that high-calorie intake disrupts lung immune homeostasis, particularly macrophage polarization. Since macrophages are critical first responders against pathogens, these changes may weaken lung defense function.

Different dietary patterns are considered important regulators of the gut microbiota and affect disease outcomes (32). Our previous study showed that a high-fat and high-calorie diet damages the gut barrier, changes the gut microbiota structure, and decreases SCFA production (13). Zhao et al. (42) found that a short-term high-fat diet changes the structure of the gut microbial community, decreases SCFA production, and is intimately associated with the occurrence and progression of mastitis. Sencio et al. (48) showed that influenza virus-induced lung infection causes gut dysbiosis in mice, decreases acetate levels, and increases susceptibility to secondary lung bacterial infection. Our study demonstrated that a high-calorie diet decreases total SCFA levels in the gut, serum, and lung tissues of LPS-induced pneumonia model mice. Interestingly, our study results showed that the distribution of different SCFAs differ in stool, serum, and lung tissues; of these, acetate is present in stool, serum, and lung tissues. This may be associated with the absorption and transport of SCFAs in the gut and the relative abundance of the gut microbiota (51–55). Stool acetate content accounts for more than 50% of total SCFAs and is the SCFA with the highest production yield (56). The concentration of circulating SCFAs is relatively low and acetate is the most abundant SCFA in peripheral circulation (57–59). Liu et al. (60) showed that SCFAs in juvenile mouse lungs originate from the gut microbiota and participate in regulating lung immune responses. Many studies have shown that acetate can be transported to the lungs and affects the function of immune cells, such as macrophages and neutrophils, to influence lung inflammation (48, 60–62). We supplemented acetate in drinking water while feeding mice a high-calorie diet in the refeeding experiment. We found that serum and lung tissue acetate levels were increased in pneumonia model mice and lung inflammatory injury was decreased, thereby proving that high-calorie diet-aggravated pneumonia is associated with decreased acetate levels.

The gut microbiota connect the gut and distal organs through the immunomodulatory effects of SCFAs. SCFAs can regulate many immune cells such as macrophages, T cells, and B cells and can affect immunophenotype and corresponding functions (63). Studies have found that acetate stimulates alveolar macrophages to produce more interferon-β so as to protect mice from respiratory syncytial virus infection, thereby decreasing the viral load and inflammatory responses (64, 65). In this study, we found that a high-calorie diet decreases gut, serum, and lung tissue acetate levels and aggravates lung tissue macrophage polarization imbalance. Acetate supplementation alleviates lung tissue macrophage polarization imbalance in LPS-induced pneumonia model mice, suggesting that acetate can regulate lung tissue macrophage polarization. However, the effects of SCFAs on macrophages are partially mediated through GPR43 activation and/or HDAC inhibition (66), yet the specific mechanisms by which SCFAs, particularly acetate, regulate macrophage polarization remain unknown. We measured GPCR and HDAC levels on the surface of lung tissue macrophages after acetate intervention and found no significant change in GPCR expression levels in lung tissue macrophages of LPS-induced pneumonia model mice after acetate supplementation; however, *Hdac9* and *Hdac10* mRNA levels were significantly downregulated, and *Hdac3, Hdac5, Hdac6, Hdac8*, and *Hdac11* mRNA levels were downregulated. Thus, the effects of acetate on HDACs in mouse lung tissue macrophages involves different types of HDACs and also suggests that the high-calorie diet-mediated acetate decrease may depend on HDAC inhibition to carry out its effects and not depend on GPR43. We further used a GPR43 inhibitor and an HDAC inhibitor for validation and found that the lung tissue macrophage phenotypic changes driven by a high-calorie diet-induced acetate decrease were not dependent on GPR43 but may be associated with HDAC inhibition. According to reports, the beneficial effects of acetate are mediated by different mechanisms, including GPCR, HDAC inhibition, and metabolism integration (22, 67). Currently, there are differing reports on the effector targets of acetate owing to different study models and experimental intervention conditions. Olaniyi et al. (68) found that acetate-mediated HDAC inhibition could alleviate streptozocin–nicotinamide-induced hepatic lipid dysregulation and its accompanying injury in diabetic rats. Soliman et al. (69) found that acetate supplementation could increase brain histone acetylation and inhibit HDAC activity and expression. Additionally, Li et al. (70) found that the GPR43 receptor does not participate in acetate regulation of macrophage activity. Our study provided evidence that acetate affects macrophage phenotype differentiation to some extent.

Macrophage metabolism has a major role in regulating macrophage phenotype and plasticity (71). M1 macrophages are highly dependent on glycolysis and M2 macrophages mainly rely on oxidative phosphorylation (35). It is worth noting that SCFAs can regulate the metabolic programming of LPS-exposed lung macrophages, which helps in maintaining lung immunometabolism (72). One study showed that SCFAs can inhibit HDAC in macrophages to promote HIF-1α expression and enhance the bactericidal activity of macrophages (73). Currently, disagreement remains over the role of HDAC in controlling HIF-1α function. Our study suggested that acetate supplementation may inhibit HIF-1α expression in lung macrophages, potentially through mechanisms involving HDAC9/10 inhibition, and was associated with reduced expression of the glycolysis-related enzyme HK2 and decreased lactic acid levels. HIF-1α can regulate the immunometabolic phenotype of macrophages (74). HIF-1α activation increases the expression of genes related to the glycolysis pathway, such as LDHA, PDK1, and HK2, and promotes glycolysis to drive M1 macrophages. This suggests that the effects of acetate on macrophage polarization might involve the inhibition of HDAC9/10, potentially influencing a downstream mechanism related to HIF-1α, HK2, and lactic acid. However, it is worth noting that we only selected HDAC9 and HDAC10, which showed significant changes after acetate intervention; the effector targets of acetate are not only HDAC9 and HDAC10, and multiple receptors may mediate changes in downstream pathways.

In conclusion, our results showed that a high-calorie diet can decrease levels of the gut microbiota metabolite acetate. This reduction in acetate was correlated with an attenuation of its inhibitory effects on HDAC9/10 and increased HIF-1α expression in lung tissue macrophages of pneumonia model mice. These changes were further associated with enhanced macrophage glycolysis and M1-like (CD206^-^CD86^+^) macrophage polarization, which contributes to aggravated lung tissue inflammatory injury. Our study results indirectly show that regulating the gut microbiota to increase SCFAs, particularly acetate, may be a strategy to prevent and treat pneumonia and other infectious diseases.

It should be noted that this study has several limitations. First, the high-calorie diet used was constructed based on previous surveys of children’s diets in China (8). The dietary components were categorized solely by broad classes such as “carbohydrates” and “crude fiber,” without more detailed nutrient comparisons. Therefore, differences in dietary components, not just caloric intake, may also influence acetate production. In future studies, we will further investigate the distinct and overlapping effects of dietary composition versus pure caloric intake. Second, we employed the M1/M2 classification for macrophages as a simplified framework to study specific functional mechanisms. Currently, there is ongoing debate regarding the M1/M2 macrophage categorization. An increasing body of literature suggests that the M1/M2 classification, originally defined based on *in vitro* experiments, is an oversimplification for such a highly plastic and heterogeneous cell type (75–77). In future work, we will continue to explore the effects of a high-calorie diet on macrophages of different phenotypes.

## Data Availability

The raw data supporting the conclusions of this article will be made available by the authors, without undue reservation.
