# Supplementary material for: A high-calorie diet exacerbates lipopolysaccharide-induced pneumonia by promoting acetate-mediated macrophage polarization via the HDAC9/10–HIF-1α–glycolysis axis
Source: Front Immunol. 2025 Sep 26;16:1614768. doi: 10.3389/fimmu.2025.1614768 (PMC12510825; doi:10.3389/fimmu.2025.1614768)
Supplement: Supplementary file 1 [file DataSheet1.docx]

**A High-Calorie Diet Exacerbates Lipopolysaccharide-Induced Pneumonia by Promoting Acetate-Mediated Macrophage Polarization via the HDAC9/10–HIF-1α–Glycolysis Axis**

**Qianqian Li ^1,2,†,^, Hui Liu ^3,†^, Chen Bai ^1^, Lin Jiang ^1^, Chen Su ^1^, Xueying Qin ^4^ ,Tiegang Liu ^1, *^, Xiaohong Gu ^1, 3, *^**

^1^ School of Traditional Chinese Medicine, Beijing University of Chinese Medicine, Beijing 102488, China

^2^ School of Traditional Chinese Medicine, Shandong University of Traditional Chinese Medicine, Jinan 250300, China

^3^ Institute of Chinese Medicine Epidemic Disease, Beijing University of Chinese Medicine, Beijing 102488, China

^4^ Dongzhimen Hospital, Beijing University of Chinese Medicine, Beijing 100700, China

**^†^** These authors have contributed equally to this work

**^*^Corresponding author：**

Tiegang Liu, School of Traditional Chinese Medicine, Beijing University of Chinese Medicine, Bei San Huan East Road, Beijing 100029, China.Email: liutiegang2009@163.com

Xiaohong Gu, School of Traditional Chinese Medicine, Beijing University of Chinese Medicine, Bei San Huan East Road, Beijing 100029, China. Email: [guxiaohong1962@163.com](mailto:guxiaohong1962@163.com)

**Table S1** The antibodies used in our study

| **Antibody** | **Vendor** | **Catalog Number** |
| --- | --- | --- |
| FITC anti-mouse CD45 | BioLegend | 103107 |
| BD Horizon™ V450 Rat anti-CD11b | BD | 560455 |
| PE/Cyanine7 anti-mouse F4/80 | BioLegend | 123114 |
| PE anti-mouse CD86 | BioLegend | 105007 |
| PE Rat IgG2a, κ Isotype Ctrl | BioLegend | 400507 |
| APC anti-mouse CD206 (MMR) | BioLegend | 141708 |
| APC Rat IgG2a, κ Isotype Ctrl | BioLegend | 400512 |
| BD Horizon™ Fixable Viability Stain 510 | BD | 564406 |
| Intracellular Staining Permeabilization Wash Buffer (10X) | BioLegend | 421002 |
| TruStain FcX™ (anti-mouse CD16/32) Antibody | BioLegend | 101320 |
| APC/Cyanine7 anti-mouse CD45 | Biolegend | 103116 |
| BV421 anti-mouse F4/80 | Biolegend | 123132 |
| FITC anti-mouse CD11b | Biolegend | 101206 |
| 7-AAD | Biolegend | 420404 |
| F4/80 | Santa | SC-377009 |
| HIF1α | CST | 36169S |
| DAPI | Beyotime | C1002 |
| GFP | Proteintech | 66002-1-Ig |

**Table S2** Experimental design of Figure1, Fugure4 and Figure 6

| **Group** | **N** | **Feed (1–6 days)** | **Atomization (4–6 days)** | **acetate(1–6 days)** | **TSA(1–6 days)** | **GLPG-0974** |
| --- | --- | --- | --- | --- | --- | --- |
| N | 6 | Mouse maintenance fodder | Physiologic saline | No | No | No |
| P | 6 | Mouse maintenance fodder | LPS solution | No | No | No |
| G | 6 | High-calorie fodder | Physiologic saline | No | No | No |
| GP | 6 | High-calorie fodder | LPS solution | No | No | No |
| Acetate | 6 | High-calorie fodder | LPS solution | Yes | No | No |
| TSA | 6 | High-calorie fodder | LPS solution | No | Yes | No |
| GLPG-0974 | 6 | High-calorie fodder | LPS solution | No | No | Yes |
| Acetate+GLPG-0974 | 6 | High-calorie fodder | LPS solution | Yes | No | Yes |
| DMSO | 6 | Mouse maintenance fodder | Physiologic saline | No | No | No |

**Table S3** Experimental design of Figure 9.

| **Group** | **N** | **AAV HDAC9** | **AAV HDAC10** | **Feed(1–6 days)** | **Atomization(4–6 days)** | **Acetate(1–6 days)** |
| --- | --- | --- | --- | --- | --- | --- |
| N | 10 | No | No | Mouse maintenance fodder | Physiologic saline | No |
| GP | 10 | No | No | High-calorie fodder | LPS solution | No |
| Acetate | 10 | No | No | High-calorie fodder | LPS solution | Yes |
| AAV HDAC9 | 10 | Yes | No | High-calorie fodder | LPS solution | Yes |
| AAV HDAC10 | 10 | No | Yes | High-calorie fodder | LPS solution | Yes |
| AAV Mock1 | 10 | No | No | High-calorie fodder | LPS solution | Yes |
| AAV Mock2 | 10 | No | No | High-calorie fodder | LPS solution | Yes |

**FigureS1**

**
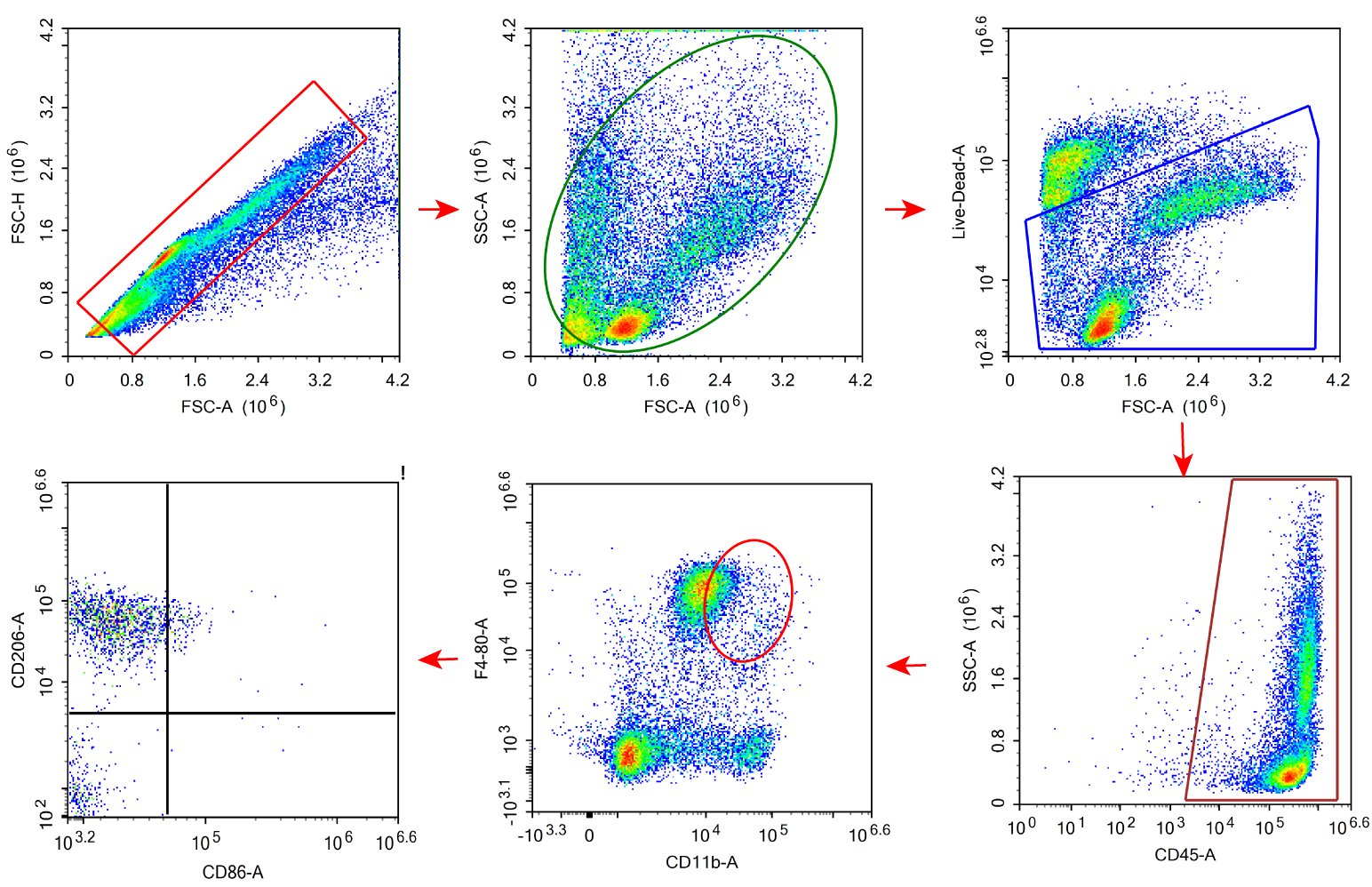
**

**FigureS1** The gating strategies for macrophages in Figure 4 and Figure 6.

**FigureS2**


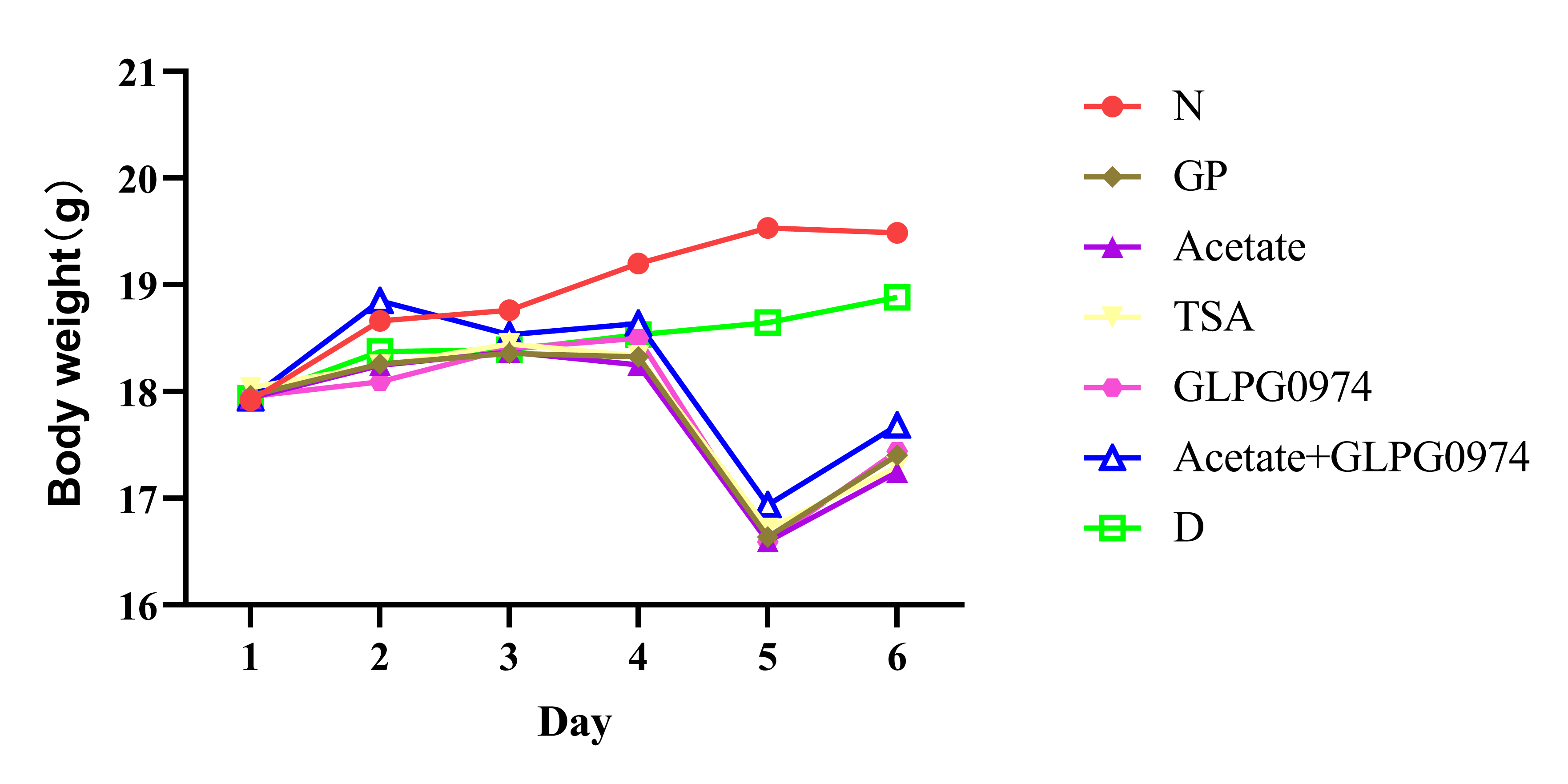


**FigureS2** Body weight changes across experimental groups.

**FigureS3**

**
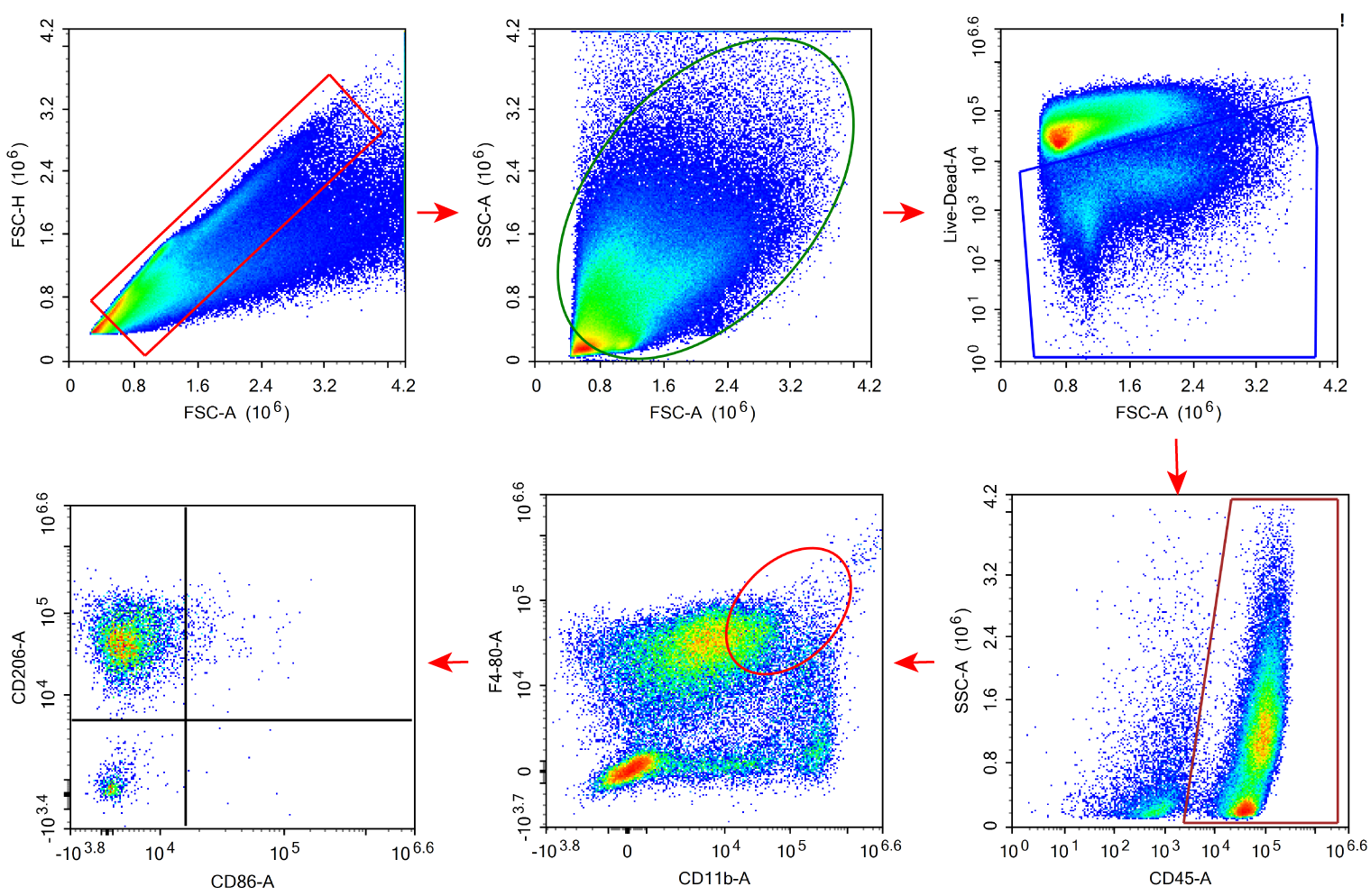
**

**FigureS3** The gating strategies for macrophages in Figure 9.
